# Characterization of spreading depolarizations in swine following superior sagittal sinus occlusion: a novel gyrencephalic model study

**DOI:** 10.1186/s12959-025-00689-w

**Published:** 2025-02-12

**Authors:** Renan Sanchez-Porras, Francisco L. Ramírez-Cuapio, Mildred A. Gutiérrez-Herrera, Ángel Alberto Puig-Lagunes, Pablo Albiña-Palmarola, Juan M. López-Navarro, Marcos Alejandro Suárez-Gutiérrez, Roberto Díaz-Peregrino, Diego A. Sandoval-Lopez, Gregor Fischer, Farzam Vazifehdan, Johannes Woitzik, Edgar Santos

**Affiliations:** 1 Department of Neurosurgery, Carl von Ossietzky University of Oldenburg, Marienstraße 11, 26121 Oldenburg, Germany; 2 Department of Neurosurgery, Heidelberg University Hospital, Ruprecht Karls University of Heidelberg, Heidelberg, Germany; 3 Faculty of medicine, Universidad Veracruzana, campus Minatitlán, Veracruz, Mexico; 4 Neuroradiologische Klinik, Klinikum Stuttgart, Stuttgart, Germany; 5 Department of Neurosurgery & Spine Center of Eastern Switzerland, St. Gallen Medical School & Cantonal Hospital of St. Gallen, St. Gallen, Switzerland; 6 Spine Center Stuttgart, Diakonie-Klinikum Stuttgart, Paulinenhilfe, Stuttgart, Germany; 7 Present Address: Department of Acute Neurology/Early Neurological Rehabilitation, Kliniken Schmieder Allensbach, Allensbach, Germany

**Keywords:** Cerebral venous occlusion, Electrocorticography (ECoG), Intrinsic optical signal imaging (IOS), Laser speckle contrast and oxygen imaging (LSCI), Spreading depolarizations (SDs), Superior sagittal sinus (SSS)

## Abstract

Cerebral sinus thrombosis, which constitutes a small percentage of all strokes, usually affects young individuals and can lead to venous stroke. Ischemic and hemorrhagic Stroke are associated with Spreading Depolarization (SD) waves in brain tissue, which propagate through the affected areas and cause a transient disruption of ionic homeostasis and neuronal function. This interaction highlights the complexity of the neurological consequences associated with SD. In this study, we investigated the occurrence of SDs following the occlusion of the superior sagittal sinus (SSS) in a gyrencephalic model, specifically swine. To instigate an occlusion, we surgically clipped the middle third of the SSS. The animals were grouped and monitored using one of three methods: electrocorticography (ECoG) alone, ECoG with intrinsic optical signal (IOS) imaging, or ECoG in conjunction with laser speckle contrast and oxygen imaging (LSCI). Post-mortem, the brains were analyzed using 2,3,5-triphenyl tetrazolium chloride (TTC) staining to check for venous infarction. Our results confirmed the spontaneous occurrence of SDs in the gyrencephalic swine brain after SSS occlusion, which was detectable via all monitoring methodologies. SD activity was most frequent in the first hour post-occlusion, subsequently diminishing. IOS imaging identified four unique hemodynamic responses, while TTC staining indicated no infarction. This research is the first to document SDs in the gyrencephalic swine brain following SSS occlusion, laying the groundwork for future investigations in both animal models and human clinical studies.

**Supplementary Information:**

The online version contains supplementary material available at 10.1186/s12959-025-00689-w.

## Introduction

Cerebral venous occlusion may result from either venous thrombosis or neurosurgical interventions, presenting with a broad spectrum of clinical manifestations and outcomes. Nevertheless, the pathophysiology and management of this condition remain poorly understood. Cerebral sinus thrombosis represents 0.5-1% of all strokes [1]. It has an estimated incidence rate of 3–4 million cases per year [2], with the majority of affected individuals being young. In a subset of patients, venous stroke may be a potential consequence. Pregnancy, history of venous thrombosis, neoplasms, Behçet’s disease, factor V Leiden mutation, prothrombin gene mutation, as well as protein C and S deficiencies, have all been identified as potential risk factors for superior sagittal sinus thrombosis [3]. Postoperative cerebral venous infarction is less well-documented but it is estimated to occur in approximately 7% of patients following major cranial surgeries [4]. Typical clinical symptoms include focal neurological deficits, signs, and symptoms of intracranial hypertension, seizure, and encephalopathy [5]. These clinical consequences underscore the necessity for a comprehensive understanding of venous ischemia. Spreading depolarizations (SDs), one of the most fundamental pathophysiological mechanisms of both pannecrotic and selective neuronal lesion development after energy deprivation, have been shown to relate to lesion progression and poor clinical outcome in cerebrovascular diseases such as stroke, subarachnoid hemorrhage, and intracerebral hemorrhage. Experimentally they are more easily induced in lissencephalic brains than in gyrencephalic brains. SDs in lissencephalic brains, for example, can be induced by mechanical stimulation following scalp exposure [6]. Lissencephalic brains exhibit reduced surface area, unequal proportions of matter, and distinctive pharmacodynamics that require the administration of significantly higher doses of SD-blocking drugs [7, 8]. In contrast, porcine brains, which are gyrencephalic in structure, are comparable to human brains. Previous experimental studies had shown similarities between the porcine brain and human recordings when studying SDs, including the types of hemodynamic responses, propagation patterns, and pharmacodynamics of SDs. Furthermore, SDs could be inhibited by drugs at doses equivalent to those used in humans [7–16]. To date, there is no direct evidence of the occurrence of SDs in the context of cerebral venous sinus thrombosis. The occurrence of SDs in cerebral venous sinus thrombosis is highly likely due to the established association between SDs and other forms of cerebrovascular occlusion. However, there is a notable lack of understanding of the characteristics of SDs following venous occlusion. To address this knowledge gap, a comprehensive investigation of the mechanisms of SDs in venous occlusion models using electrocorticography (ECoG) and other advanced monitoring techniques is needed. In addition, a model that accurately reflects the structural and functional characteristics of the human cerebral cortex is essential for the study of SDs. The porcine brain, with its gyrencephalic structure, provides a closer approximation to the human brain than rodent models, particularly in the propagation of SDs across cortical regions. The present study aimed to advance our understanding of SDs in the context of cerebral venous occlusion, focusing on the characterization of the hemodynamic response by using intrinsic optical signal (IOS) imaging and laser speckle contrast imaging coupled with oxygenation assessment (LSCI) in a gyrencephalic model to characterize the hemodynamic response. Our approach was designed to provide new insights into the occurrence of SD after superior sagittal sinus (SSS) occlusion that can be translated to human pathology.

## Materials and methods

All experiments were performed in accordance with the German Animal Welfare Act (Tierschutzgesetz). The animal protocol for the experiments (nr. 35-9185.81/G-60/19) was approved by the Institutional Animal Care and Use Committee (Karlsruhe, Baden-Württemberg, Germany). This reporting complies with ARRIVE (Animal Research Reporting In Vivo Experiments) Guidelines.

### Experimental groups

**Fig. 1:**
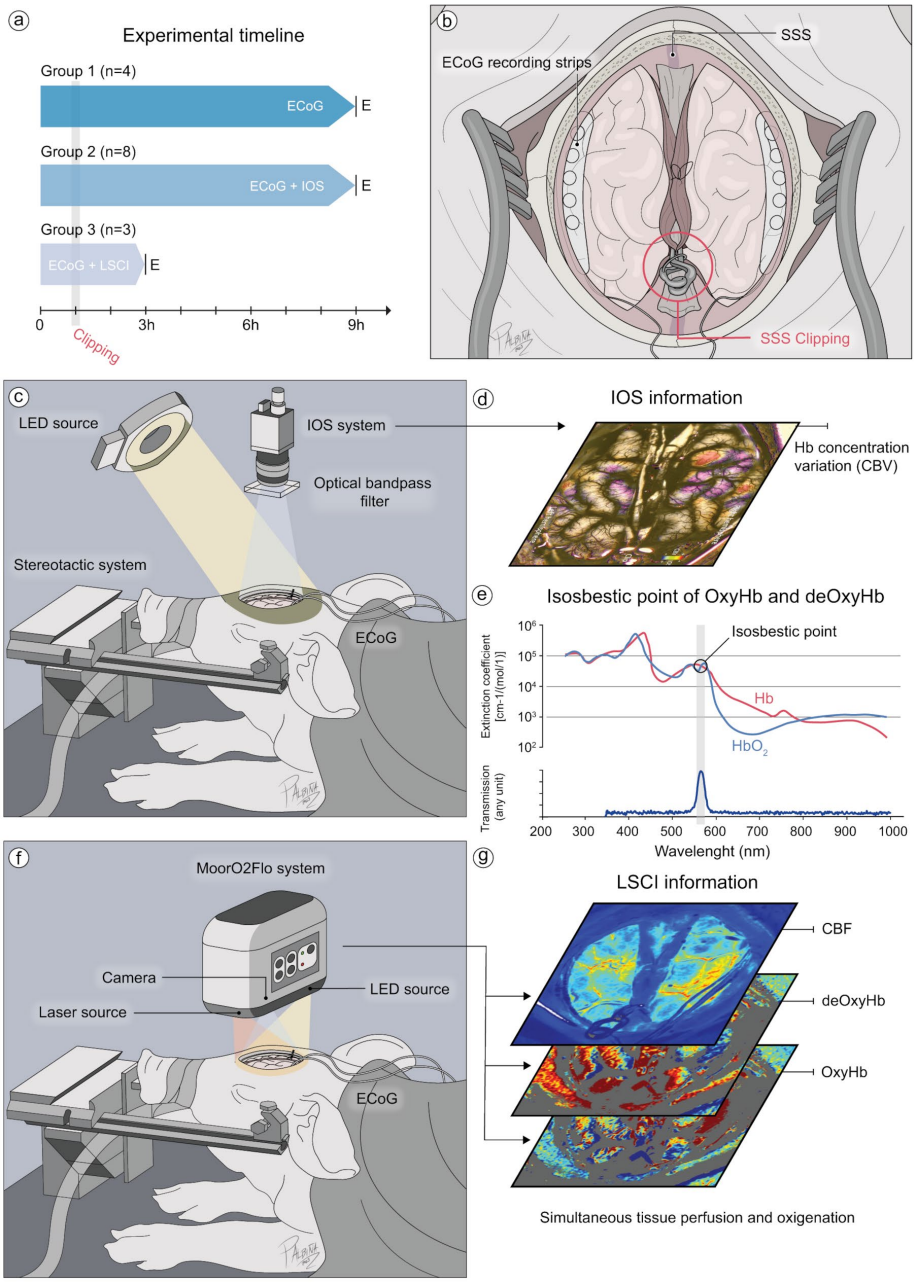
Overview of study design and the experimental setup. **a**) A total of 15 swine were categorized into three distinct groups based on the modality used for SD detection: (1) ECoG (*n* = 4), (2) ECoG + IOS (*n* = 8), or (3) ECoG + LSCI (*n* = 3). (**b**) To evaluate the spontaneous induction of SD after venous occlusion, a surgical clipping of the middle third of the SSS was performed. (**c**) Simultaneous recordings of ECoG and IOS were made in the second group. (**d**) Regional hemoglobin concentration variations in the cerebral cortex were considered as a surrogate of CBV. An optical band-pass filter (564 nm) was positioned in front of the lens of the IOS system, enabling us to detect the (**e**) isosbestic point of OxyHb, and deOxyHb. (**f**) Simultaneous recordings of ECoG and LSCI were performed using the MoorO2Flo system to evaluate perfusion and oxygenation changes in the third group. (**g**) Data obtained through this recording system allowed us to analyze variations in CBF, OxyHb, and deOxyHb during SD development. CBF: cerebral blood flow; CBV: cerebral blood volume; deOxyHb: deoxyhemoglobin; E: end (of recording); ECoG: Electrocorticography; IOS: Intrinsic optical signal; LED: light emitting diode; LSCI: laser speckle contrast and oxygen imaging; OxyHb: oxyhemoglobin; SSS: superior sagittal sinus

This study incorporated a total of 18 swine. Three animals were utilized for surgical training and method standardization. The remaining 15 animals were divided into three experimental groups (Fig. 1a). In group 1 (*n* = 4), animals were used to evaluate the feasibility of detecting SDs after SSS occlusion using ECoG alone, with monitorization performed for up to 9 h. In group 2 (*n* = 8), animals underwent combined monitoring utilizing ECoG and IOS imaging for the same duration. Finally, in group 3 (*n* = 3), a simultaneous recording with ECoG and LSCI (MoorO2Flo, Moor instruments, Devon, UK) was performed for up to 3 h.

### Animal preparation

Female German Landrace swine, aged 3–4 months and weighing 29–36.5 kg, were used. All the animals were anesthetized intramuscularly with midazolam (Dormicum^®^, 0.5–0.7 mg/kg) and azaperone (Stresnil^®^, 4 mg/kg), followed by an intravenous propofol 2% bolus of 40 mg (Disprovan^®^, 1–2 mg/kg) immediately prior to intubation. Animals were then intubated and mechanically ventilated under anesthesia. Maintenance of anesthesia included inhaled isoflurane (Isothesia^®^) at 0.6–1.0% and midazolam perfusion. A venous line was placed in the right ear vein. Peripheral capillary oxygen saturation (SpO_2_) and heart rate (HR) were monitored from the left ear. After surgical exposure of the right femoral artery, a 4-Fr catheter was inserted to continuously monitor the mean arterial pressure (MAP) (Raumedic AG, Helmbrechts, Germany). Rectal temperature was continuously measured during the experiment using a temperature sensor. Cardiovascular and respiratory monitoring was performed as previously described [9]. The HR was maintained between 60 and 120 bpm, MAP between 65 and 100 mmHg, SpO_2_ at > 90%, pCO_2_ at 35–45 mmHg, pO_2_ at > 80 mmHg, and temperature 36–38 °C. After finishing the experiment, animals were euthanized with an IV overdose of KCl under anesthesia.

### Operative procedures

A craniotomy was performed to expose the SSS. For this purpose, the heads of animals were kept in a prone position, with the head firmly held in a custom-made stereotactic frame. The area of the craniotomy was marked on the scalp according to the following orientation marks: 1 cm posterior to the line of the eyes, 0.5 cm in medial direction of the orbits, and 0.5 cm frontal to the occipital protuberance. A midline skin incision was made, and the scalp was retracted. This was followed by bilateral burr holes over the parietal bones; thereafter the craniotomy was performed using a high-speed drill under cooling with saline solution. The bone flap was carefully removed. The dura mater was then incised bilaterally to reveal the hemispheres, finally the superior sagittal sinus was exposed. Two 5-contact ECoG strips (Ad-Tech Medical Instrument Corp., Racine, WI, USA) were positioned on both cerebral hemispheres for recording purposes. After one hour of baseline ECoG measurements, a surgical clip (Aesculap^®^, Yasargil’s temporary clip, 7.0 mm, FT240T) was applied to the middle third of the SSS to induce occlusion in groups 2 and 3 (Fig. 1b). The setups for IOS imaging and LSCI using the MoorO2Flo device were positioned over the cortical surface (Fig. 1c and f). The exposed cortex was protected with a paraffin pool (1–1.5 cm) to minimize light reflection and cover the entire cortex. The paraffin pool was refreshed, and new paraffin was added to maintain image acquisition quality. Occlusion was performed, as mentioned above, by clipping the middle third of the SSS. During the occlusion, real-time visual inspections of the SSS were performed to detect immediate thrombus formation. Imaging techniques, IOS and LSCI, were used to monitor the hemodynamic changes after clipping. This would allow us to track the hemodynamic changes after thrombosis or infarct progression and assess their impact on CBV and CBF. Additionally, we performed pathological post-mortem analysis as well as histological analysis of tissue samples to examine thrombus formation.

### Monitoring of spreading depolarization

*ECoG monitoring*. ECoG electrodes were connected sequentially in a monopolar arrangement. The monitoring’s sampling rate was set to 1,000 Hz, utilizing a PowerLab 16/SP near-DC amplifier coupled with an analog-to-digital converter. The ECoG data was recorded using LabChart 7 software (AD Instruments, Bella Vista, Australia). ECoG recording continued throughout the duration of IOS and LSCI monitoring to ensure continuous data collection.

*IOS monitoring*. IOS imaging was executed using a charge-coupled device (CCD) camera (Smartec GC1621M 8-bit grayscale, 1628 × 1236 pixels, 1/1.8 sensor; MaxxVision GmbH, Stuttgart, Germany). The camera, fitted with a 50 mm lens (Fujinon HF50HA-1B 50 mm Fixed Focal Lens, f = 1.4; MaxxVision GmbH), was positioned 25–30 cm above the exposed brain surface. An optical band-pass filter (564 nm, 14 nm FWHM, Schott, Germany) was installed before of the lens, which selectively transmitted green spectrum light. The reflected light mainly indicated the total hemoglobin concentration in the tissue. Images were captured at a rate of one per second at full resolution. Post-acquisition, the images underwent an elastic transformation as part of an offline registration process, enhancing the quality of imaging and analysis, as applied in previous studies [10, 14, 16].

*LSCI monitoring*. The laser was placed about 20–30 cm above the exposed cortex. The procedure adhered to the manufacturer’s guidelines for data acquisition (Moor Instruments, Devon, UK). The device setup was as follows: Model MoorO2Flo (SN3), camera gain set to 125, and an exposure time of 20 ms. The imaging was configured in Spatial mode with a kernel size of 5 × 5, a time contrast setting of 1.0 s, and a sampling interval of 5000 ms. Images were captured at a resolution of 748 × 576 pixels.

### Data analysis

*ECoG analysis*. ECoG monitoring was performed in all groups. Recordings were analyzed for SD events following the recommendations of the Co-Operative Studies on Brain Injury Depolarizations (COSBID) ECoG signals [17]. All ECoG files were analyzed using LabChart 7 (AD Instruments, Bella Vista, Australia) software. SDs from the ECoG files were analyzed for incidence (SDs/h) and expansion, which was expressed as the percentage (%) of the total number of ECoG channels in which SDs were detected.

*IOS analysis*. IOS was performed in group 2. Images needed to be post-processed to enable their offline analysis. An elastic registration algorithm was performed to compensate for movement artifacts due to animal breathing, shivering, and brain pulsations. Data were excluded if there were strong movements that could not be compensated by our algorithm. Ten to fifteen regions of interest (ROIs) of approximately 5 pixels (0.03–0.12 mm2) were distributed symmetrically in the left and right hemispheres. Because IOS enables the visualization of regional hemoglobin concertation changes in the cortical tissue, it was used as a surrogate of regional cerebral blood volume (CBV) changes (Fig. 1d and e). Thus, increases in IOS intensity will represent a decrease in hemoglobin tissue concentration (hypoperfusion or oligemic responses), while decreases in IOS intensity will represent an increase in hemoglobin concentration (hyperemic responses). Intensity profiles extracted from ROIs during SD were analyzed using custom-developed software based on ImageJ [14]. SD was identified as an intensity change or changes, expanding to at least two ROIs. Intensity changes could be simultaneously plotted and visualized in our software. SDs obtained from the IOS files were analyzed for incidence (SDs/h) and expansion, defined as the number of ROIs reached in which one SD is detected in the visible area, expressed in percentage (%). The morphology of each SDs was defined according to the amplitude fluctuations of the hyper- or hypo-intensity changes after an individual baseline was established. Differences in amplitude and duration of the hyperemic response between the first and last SDs detected were analyzed per ROI in all animals who presented a minimum of two SDs within the same hemisphere during the 9 h of recording time. However, it is essential to note that IOS imaging intensities can be influenced by various factors that cannot be fully eliminated without measuring additional wavelengths or using other technique modalities [18, 19].

*LSCI analysis*. This method was performed in group 3 to obtain perfusion and oxygenation data during SD development. Analysis was achieved using the manufacturer’s software. Data were excluded if strong movements were present. Four ROIs of about 0.7 mm^2^ (0.8 mm X 0.8 mm) were distributed symmetrically in the left and right hemispheres. Cerebral blood flow (CBF) data, oxyhemoglobin (OxyHb), and deoxyhemoglobin (deOxyHb) were extracted for the analysis (Fig. 1g). Data were expressed in arbitrary perfusion units (APU). Due to the nature of the study and the data availability in a small group of animals, only a descriptive and not statical analysis was performed for LSCI.

### Infarct volume measurement

Ischemic infarct volume was estimated via 2,3,5-triphenyl tetrazolium chloride (TTC) staining. This was performed in groups 1 and 2. After termination, the brains were rapidly removed, placed in a mold of dental alginate, semi-frozen at -20 °C for approximately 10 min, and coronally sectioned into 5-mm-thick slices using a custom-made cutting device. The brain slices were then incubated with 2% TTC in NaCl for 30 min at 37 °C. TTC-stained brain sections were arranged in frontal to occipital orientation. The brain slices were fixed in a 4% formalin solution for five days. After fixation, all brain slices were scanned using a flatbed scanner (Canon CanoScan LiDE, Japan) on both sides. Each brain slice’s unstained area (TTC negative) was defined as an infarction. Stained and non-stained regions were measured using ImageJ version 1.44p (National Institutes of Health, Bethesda, MD, USA).

### Statistical analysis

Descriptive statistics were calculated for all outcome variables of interest. Incidence and expansion comparisons between the first and second hours were performed using the Wilcoxon signed-rank test. Differences in incidence and expansion between hemispheres and the difference in amplitude and duration of the hyperemic responses to SD were performed using the Mann-Whitney U test. Tests were chosen based on the normality and homoscedasticity of variance. The results are reported as means and standard deviations (STD ±). The confidence interval (CI) was 95%. A *p*-value less than 5% (*p* < 0.05) was considered statistically significant. Given that all analyses were exploratory, no correction for multiple testing was conducted. Statistical analyses were performed using SPSS software version 19 (SPSS Inc., Chicago, IL, USA).

## Results

### Physiological parameters

Systemic physiological parameters were continuously monitored during experiments and kept within the normal range (Table 1).

**Table 1:** Physiological parameters

Physiological parameter	Values	
	Mean	STD
Weight (kg)	31.6	2.5
Heart rate (beats/min)	107.4	15.3
MAP (mmHg)	77.6	8.3
SpO_2_ (%)	99.6	1.0
pCO_2_ (mmHg)	41.7	2.5
Rectal temperature (°C)	36.5	0.4

Values are expressed in mean and standard deviation (STD ±). MAP: mean arterial pressure; SpO_2_: peripheral capillary oxygen saturation; pCO_2_: partial pressure of carbon dioxide

### Monitoring after superior sagittal sinus occlusion

All groups were prospectively monitored with ECoG following SSS occlusion for a total monitoring time of 105.0 h with a mean of 7.0 h (STD ± 2.8) per experiment. In group 1, the mean duration of ECoG recording was 7.0 h (STD ± 1.4); in group 2, 8.4 h (STD ± 2.3); and in group 3, 3.2 h (STD ± 2.2). Due to longer monitoring information, only ECoG data from groups 1 and 2 were used for SD analysis. For IOS data, the recordings of two animals were excluded due to non-compensable movement artifacts after registration, leaving six animals for analysis. The total monitoring duration of IOS recordings for analysis was 38.3 h, with a mean of 6.4 h (STD ± 3.7) per experiment. In the LSCI data set, recordings from only one animal were viable for analysis, as 2 animals had to be excluded due to non-compensable movement artifacts. The total duration of the LSCI recording was 2.5 h.

### Spreading depolarization after superior sagittal sinus occlusion

**Fig. 2:**
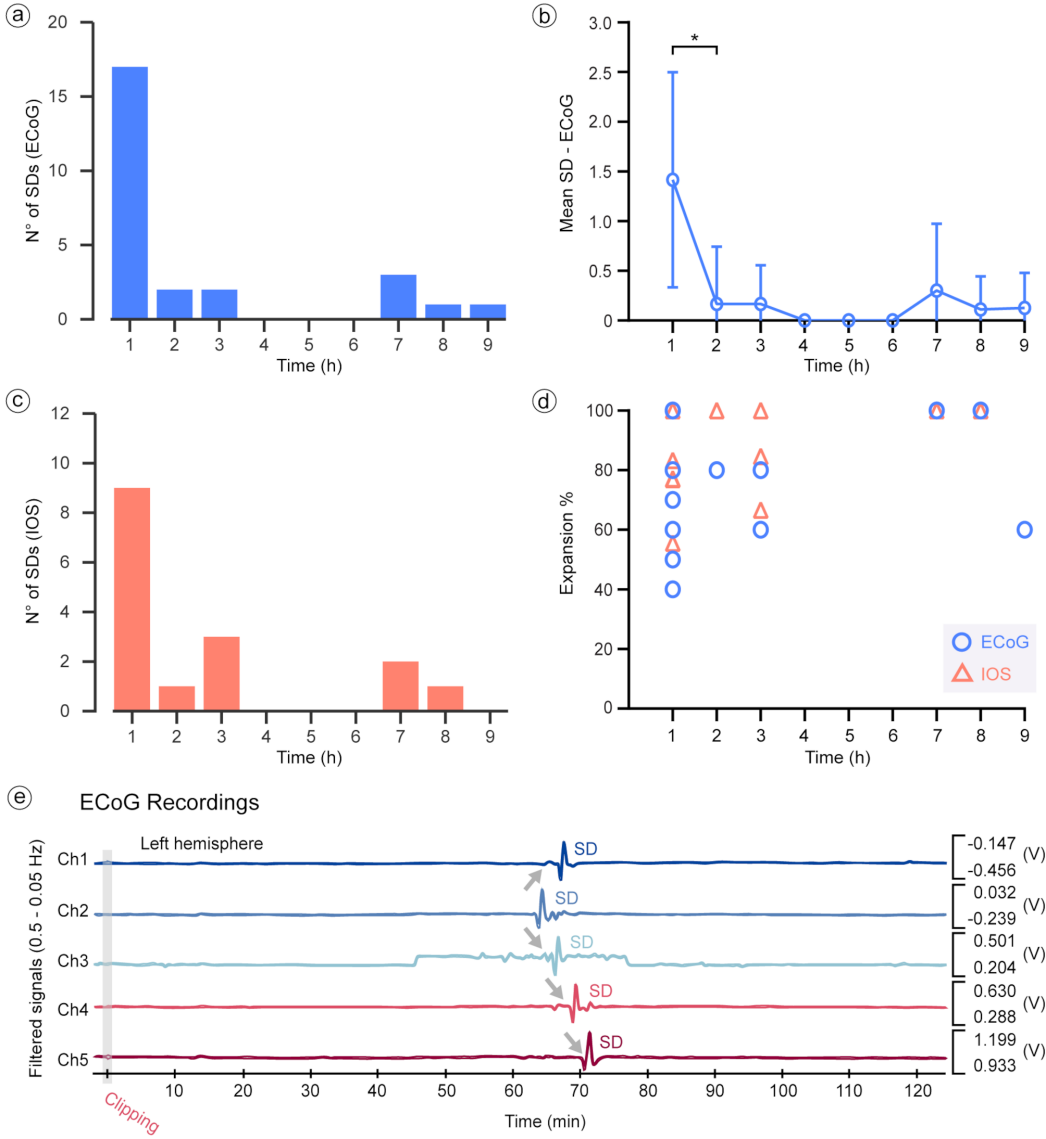
SD incidence and expansion as monitored by ECoG and IOS. (**a**) The count of SDs recorded across groups 1 and 2 is visualized in bar graph format, highlighting that a majority of SDs were identified within the initial hour subsequent to SSS occlusion, with a notable decrease in the ensuing hours. Interestingly, a transient SD-free interval was observed between the fourth and sixth hours post-occlusion. (**b**) Examination of SD occurrence in the ECoG dataset and (**c**) verification in the IOS dataset both substantiate a decremental trend in the average frequency of SDs over the duration of the study, with a statistically significant diminution from the first to the second-hour post-occlusion as evidenced in ECoG (*p* = 0.014) and IOS (*p* = 0.038) recordings. (**d**) The analysis of SD expansion metrics, ascertained through both ECoG and IOS, revealed a marked variability initially, which stabilized over time. This variability was not statistically significant when comparing the first hour to the subsequent hour. (**e**) Representative traces of SD activity detected via ECoG within the initial two hours post-occlusion are provided for illustrative purposes. ECoG: electrocorticography; IOS: intrinsic optical signal; SD: spreading depolarization; V: volts

The mean time to the onset of the first SD after SSS occlusion in the analyzed ECoG recordings was 49.3 min (STD ± 116.4). For IOS, the mean time to the onset of the first SD detected after the sinus occlusion was 101 min (STD ± 136). In one of the six animals, a transient increase in CBV was indicated by a slight decrease in the IOS intensity signal immediately after clipping, which then normalized to baseline values. After SSS occlusion, a total of 26 SDs (Fig. 2a) were detected in ECoG during the 95.3 h of recordings analyzed in groups 1 and 2 (Fig. 2e). SDs developed in 10 out of 12 animals, whereas in two swine, no SDs were detected during the monitoring time. The overall incidence rate of the detected SDs was 0.3 SDs/h (STD ± 0.7) during the total monitoring time. During the first hour, SDs were detected in 75% (9/12) of the animals. A total of 17 SDs were identified during this period, with a mean of 1.4 SDs (STD ± 1.1) per animal. In comparison, a mean of 0.2 SDs (STD ± 0.6) per animal was observed during the following hour. The first hour showed therefore a significantly higher incidence of SDs in comparison to the second hour (*p* = 0.014) (Fig. 2b). SD incidence was observed to decline in the subsequent hours, with no further development of SD between the fourth and sixth hours after the clipping. The mean expansion per animal observed in the ECoG data was 69.4% (STD ± 22.6) during the monitoring period (Fig. 2d). No statistically significant difference was observed between the first and second hours for expansion (*p* = 0.317). Analysis of the difference between hemispheres also showed no significant difference between the right and left hemispheres for either incidence (*p* = 0.143) or expansion (*p* = 0.211) during the monitoring period. In IOS, in group 2, a total of 16 SDs were identified after SSS occlusion during the total monitoring time (Fig. 2c). Consistent with the ECoG findings, IOS monitoring revealed an increased total number of SDs development within the first hour after occlusion. During the first hour, SDs were detected in 83.3% (5/6) of the animals. A total of 9 SDs were identified during the first hour, having an incidence of 1.5 SDs (STD ± 0.8) per animal. In contrast, during the second hour, only one SD was detected in one animal. Therefore, an analysis of the mean incidence between the first and second hour of monitoring was not possible. However, a significant difference was found when comparing the total number of SDs between the first and second hours of monitoring (*p* = 0.038). Similar to the ECoG results, a decrease in the total number of SDs was observed over subsequent hours, with no SD events detected between the fourth and sixth hours. The mean expansion per animal observed in the IOS data was 85.8% (STD ± 15.7) over the monitoring period (Fig. 2d). No significant difference was observed when comparing the SD expansion between the first and second hour (*p* = 0.317). Furthermore, no significant differences were observed between the total number of SDs (*p* = 0.589) or the expansion (*p* = 0.818) when comparing the right and left hemispheres.

### Hemodynamic response of spreading depolarization after superior sagittal sinus occlusion

**Fig. 3:**
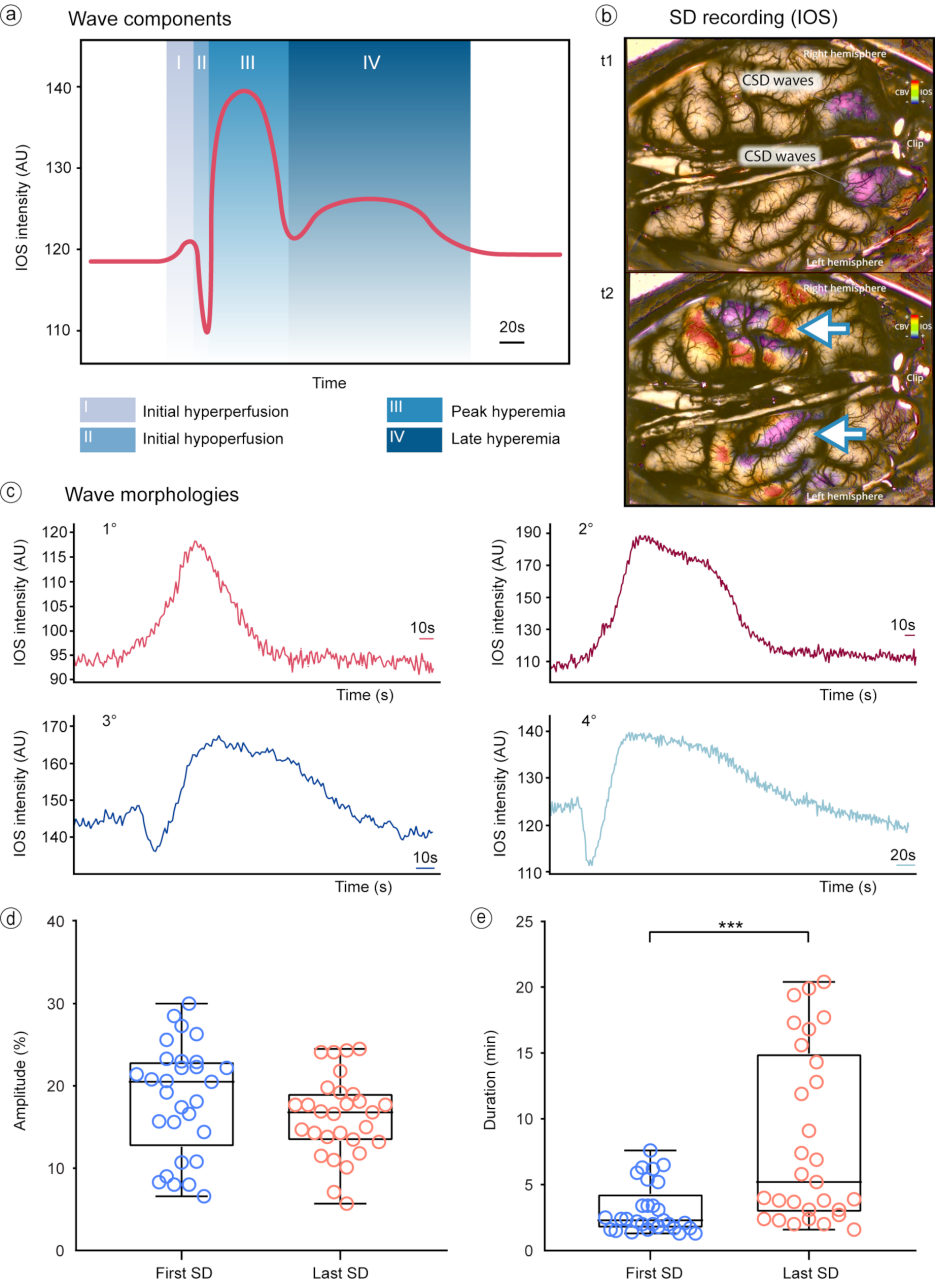
Analysis of hemodynamic changes associated with SD induced by SSS occlusion as detected by IOS. (**a**) Within the IOS recordings, we delineated four distinct phases constituting the prototypical hemodynamic responses to SD. (**b**) A segment from an IOS recording session is presented, illustrating the propagation of SD across the cortical surface of both cerebral hemispheres. Notably, the dynamic changes in CBV are captured and highlighted through IOS imaging techniques. (**c**) Subsequent to the assessment of hemodynamic responses elicited by SD episodes, we characterized four discrete waveforms, each showcasing a hyperemic response as the predominant feature across the observed morphologies. (**d**) A comprehensive evaluation of the amplitude and (**e**) the duration of these hyperemic responses for each ROI from the initial to the final SD, revealed a significant prolongation in duration (*p* < 0.001), whereas alterations in amplitude did not achieve statistical significance. Each circle in the box plot represents a data point of mean amplitude/duration for each analyzed ROI during the first (blue) or last (red) SDs events. AU: arbitrary units; CBV: cerebral blood volume; IOS: intrinsic optical signal; ROI: region of interest; SD: spreading depolarization; SSS: superior sagittal sinus

IOS monitoring revealed diverse hemodynamic responses as SDs propagated through the various ROIs following superior sagittal sinus occlusion. Within these hemodynamic responses, we identify some of the SD vasomotor components previously described in the literature, namely an initial hyper/hypoperfusion, a peak hyperemia, and a late hyperemia, as shown in Fig. 3a. Video 1 (Online Resource 1) shows an example in IOS of an SD propagating through the cerebral cortex after SSS occlusion, where SDs are evident as changes in intensity across the cortex (Fig. 3b).

Four primary types of hemodynamic responses or morphologies associated with SDs were characterized (Fig. 3c):

1. A monophasic response characterized by peak hyperemia (component III).
2. A biphasic response, which included peak hyperemia (component III) followed by late superimposed hyperemia (component IV).
3. A tetraphasic response, encompassing a brief hyperemic phase (component I), initial hypoperfusion (component II), followed by peak hyperemia (component III), and concluding with late hyperemia (component IV).
4. A triphasic response involving initial hypoperfusion (component II), followed by peak hyperemia (component III) and then late hyperemia (component IV).

It is worth noting that at least two of these morphologies were observed within each animal during the IOS recording period. Additionally, an analysis of all ROIs, particularly those nearest to the occlusion site in both the left and right cerebral hemispheres, revealed that the most prevalent responses were the monophasic peak hyperemia and the biphasic response with superimposed hyperemia, each accounting for 31.2% of the hemodynamic patterns observed.

### Amplitude and duration of the hyperemic response to spreading depolarization over time

Given the prevalence of hyperemic responses to SD, either in isolation or preceding a late response, we focused on the amplitude and duration of these responses. Our analysis covered the first and last SD events for every ROI within the same hemisphere across 9 h of post-SSS occlusion recording. Amplitude changes did not show a significant statistical difference, with mean values of 18.5% (STD ± 6.7) for the first SD and 16.3% (STD ± 4.9) for the last SD (*p* = 0.140), as depicted in Fig. 3d. However, a significant difference in the duration of these events was observed (*p* < 0.001), with a mean duration of 3.1 min (STD ± 1.9) for the first SDs and a mean duration of 8.3 min (STD ± 6.6) for the last SDs, as shown in Fig. 3e. A greater variability in the distribution of data regarding the duration of the SD was observed for the last SDs compared to the first SDs, as illustrated in Fig. 3e.

### Spreading depolarization detected with laser speckle contrast and oxygen imaging

**Fig. 4:**
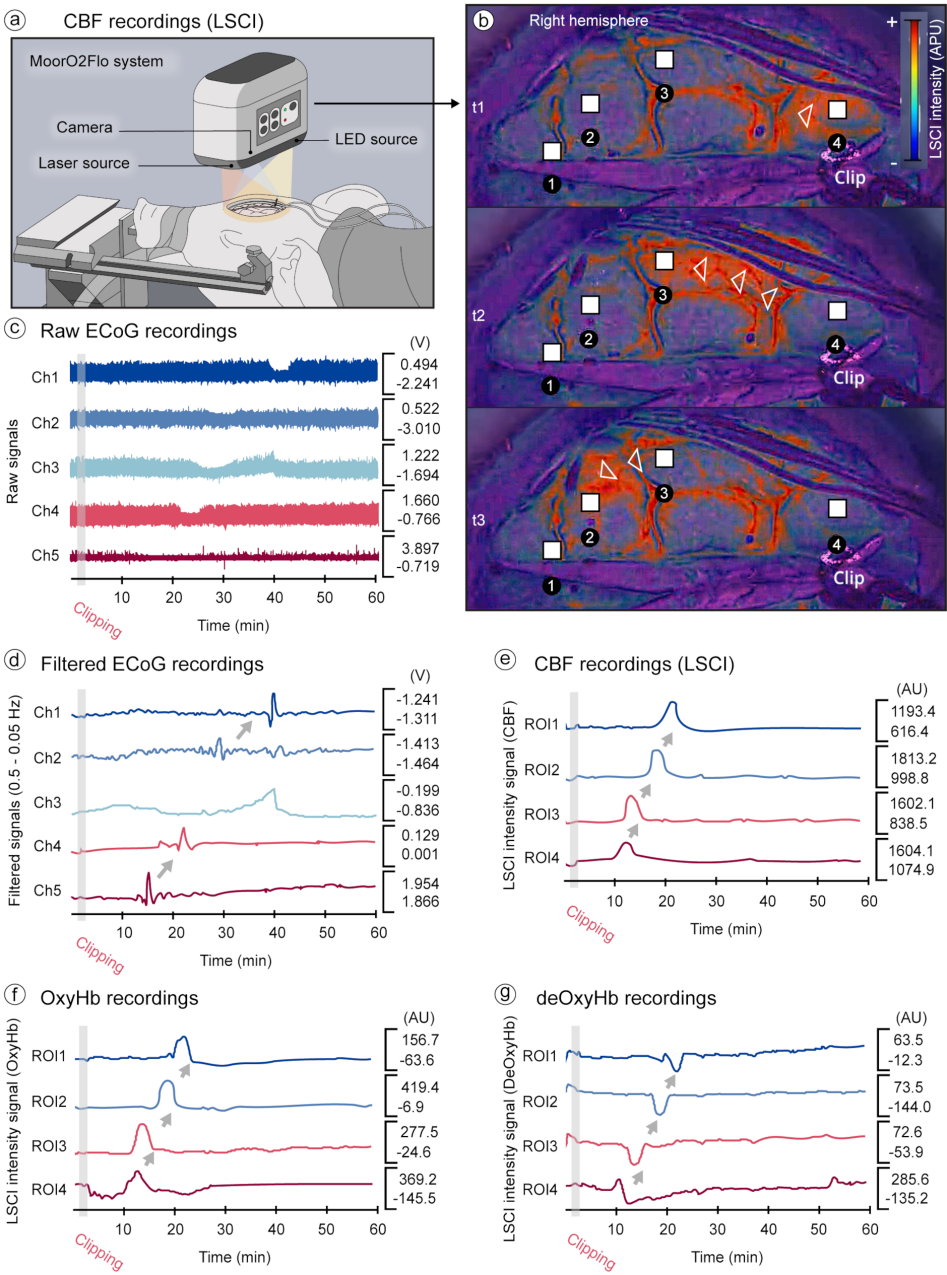
Characterization of cerebral hemodynamic response to SD post-SSS occlusion via LSCI. (**a**) The utilization of the continuous LSCI methodology through the MoorO2Flo system allowed SD detection. (**b**) Specifically, the emergence of an SD was recorded at nine minutes subsequent to the occlusion of the SSS. This SD exhibited directional propagation through the right hemisphere of the cerebral cortex, progressing from the parietal (ROI 4) to the frontal (ROI 1) regions, with the point of origin being the site of SSS clipping. The variations detected in LSCI signal intensity reflect corresponding changes in CBF in response to the SD. This hemodynamic pattern was temporally aligned with the detection of SD on concurrent ECoG recordings, captured as (**c**) unprocessed signal tracings, and (**d**) through signals subjected to low-pass filtering (0.5 –0.05 Hz), facilitating clearer visualization of slow-frequency events. (**e**) Subsequent to the occlusion of the SSS, a uniform monophasic increase in CBF, indicative of transient hyperemia, was observed across four predefined ROIs within the right cerebral hemisphere. (**f**) and (**g**) Further analyses delineating the OxyHb and deOxyHb responses uncovered diverse waveform morphologies corresponding to the SD’s trajectory across the four designated ROIs on the right hemisphere of the cerebral cortex. AU: arbitrary units; CBF: cerebral blood flow; deOxyHb: deoxyhemoglobin; ECoG: electrocorticography; LED: light emitting diode; LSCI: laser speckle contrast and oxygen imaging; OxyHb: oxyhemoglobin; ROIs: regions of interest; SSS: superior sagittal sinus

Due to technical limitations and movement artifacts, LSCI data could only be reliably collected from a single subject. Consequently, these findings are considered descriptive and exploratory. Following SSS occlusion, LSCI detected one SD on the right hemisphere, occurring at 9 min post-occlusion (Fig. 4a and b), which corresponded chronologically to the detection of the same SD in the ECoG recorded at 12 min (Fig. 4c and d). As the SD evolved, there was a prompt and synchronous rise in CBF and OxyHb levels, moving from the parietal (ROI 4) to the frontal region (ROI 1) adjacent to the occlusion site, followed shortly by a decrease in deOxyHb. These dynamics are captured in Videos 2a-c (Online Resource 2). Analysis of the four ROIs on the right hemisphere revealed a monophasic CBF pattern (Fig. 4e) characterized by a transient elevation of 61.5% (STD ± 19.7), subsequently returning to baseline. In concordance, there was a corresponding rise in OxyHb alongside a fall in deOxyHb as the SD traversed these ROIs (Fig. 4f and g).

### TTC staining and histological infarct inspection

**Fig. 5:**
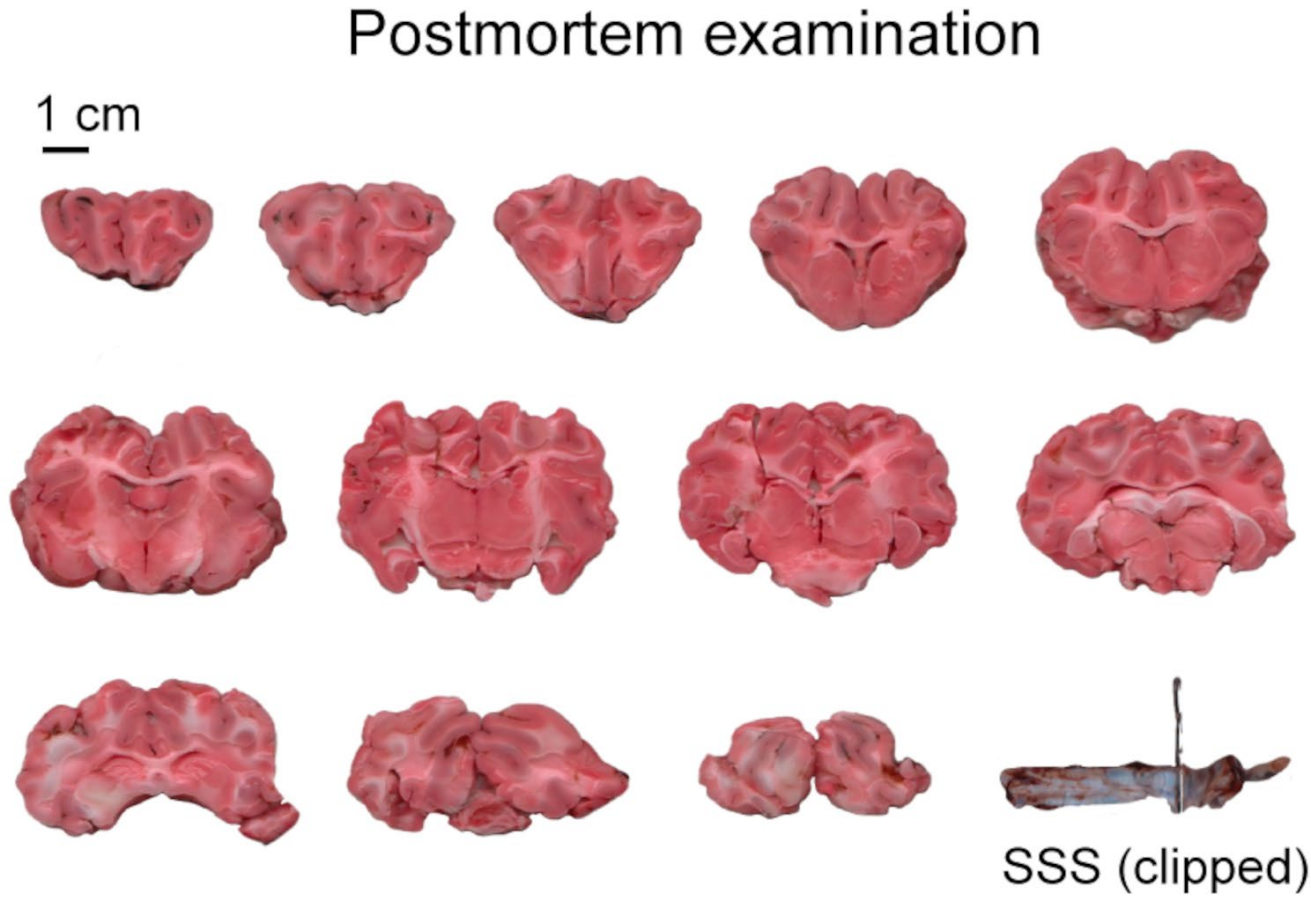
Assessment of cerebral infarction via TTC staining in postmortem analysis. The application of 2,3,5-triphenyl tetrazolium chloride (TTC) staining to 5-mm coronal sections of brain tissue facilitated the evaluation for ischemic damage. Analysis performed 9 h post-occlusion of the Superior Sagittal Sinus (SSS) revealed no discernible evidence of infarcted brain tissue

Upon conducting post-mortem TTC staining on brain sections from both experimental groups (groups 1 and 2), we observed an absence of infarct across all 5-mm brain slices and within the SSS itself (Fig. 5). A comprehensive microscopic examination of the SSS revealed that, following an average of 7.9 h (STD ± 2.4) between SSS occlusion and animal sacrifice in both experimental groups, there was no evidence of thrombus formation.

## Discussion

This study demonstrates that SDs can occur following cerebral venous occlusion in the gyrencephalic brain. We observed that SDs can occur after clipping the middle third of the SSS in the swine brain. SDs were monitored using a combination of ECoG, IOS imaging, and LSCI, all well-established techniques for SD detection. Our findings showed that SDs occurred with the highest frequency during the first hour post-occlusion, with a gradual decline in incidence over time. Notably, no SDs were detected between the 4th and 6th hours post-occlusion. There was no difference in SD expansion or incidence between hemispheres. IOS imaging allowed us to distinguish different SD hemodynamic responses and components. Among the four identified types of hemodynamic responses, hyperemia was the most common. While the amplitude of this hyperemic response remained constant, its duration increased over time. LSCI provided valuable visualization of SD propagation, showing expansion from the parietal to the frontal regions, correlated with changes in CBF and oxygenation. Finally, post-mortem TTC staining revealed no evidence of thrombus formation or venous infarction during the monitoring period. Over the past twenty years, extensive literature substantiates the occurrence of SDs within the human cerebrum, implicating their significance in cerebrovascular pathologies. Numerous studies delineate a correlation between SD episodes and neuronal functional impairment, neurodegeneration, and suboptimal clinical outcomes [20–25]. Notably, the demonstration of SDs post-venous occlusion within a translational gyrencephalic model underscores their potential clinical significance. Cerebral venous sinus thrombosis is a rare type of stroke. Similarly, instances of postoperative venous occlusion can occur, and in certain cases, occlusion of the SSS may be necessary. In both scenarios, clinical manifestations differ from those typically observed in arterial strokes. Clinical presentations are heterogeneous, with a spectrum ranging from asymptomatic cases to transient neurological deficits, and in a subset, culminating in irreversible disability or mortality [26]. SDs may partially elucidate the transient neurological impairments observed in these cases. Notably, SSS occlusion frequently precipitates epileptogenic phenomena [27–29]. While epileptic discharges have the propensity to trigger SDs, evidence suggests SDs concurrently possess an inhibitory effect on seizure propagation by hindering neuronal synchrony and interrupting epileptiform cascades [30–32]. This dichotomy posits a potentially protective role for SDs in mitigating epileptiform propagation across cortical strata and stymying widespread seizure activity. Therefore, investigations using suitable translational models are indispensable to ascertain the precise clinical effects of SDs in the context of cerebral venous occlusive phenomena. Most of the experimental studies of venous occlusion have been carried out in rodents. However, the translation of findings from lissencephalic rodents to gyrencephalic human brains is complex [33]. Our choice of a swine model is justified by its closer resemblance to the human cerebrum. Translational swine models of experimental sinus occlusion using different techniques such as endovascular flow blockage, thrombin injection, distal thrombus formation, and photothrombosis have been previously described [34–38]. They report occlusion-dependent hemodynamic changes ranging from a well-tolerated partial thrombosis to a complete thrombosis, where no collateralization is possible, and successive infarct formation [34–38]. In our model, with the exception of one animal, we did not observe an apparent hemodynamic and perfusion change in IOS imaging immediately after clipping. Neither were we able to document venous infarction development, at least during the monitoring time. This would speak in support of a well-tolerated occlusion with possible collateral out-flow development.

Anatomical differences and changes of out-flow due to possible vein collateralization after the sinus occlusion might help to explain our present findings. Unlike humans, the venous system is configured differently in swine, which might account for the absence of venous infarction post-SSS occlusion attempts in previous studies [9]. Besides, in swine, the sagittal sinus communicates with the confluence sinus and transverse sinuses, with the system draining into the spinal epidural venous plexus. In comparison, in humans, the spinal venous plexus connects with the internal jugular vein [34, 38], and the occlusion of the first third of the SSS generally causes no clinical problems [39]. However, the occlusion of the mid-SSS, as was done in the current study, or posterior third, is not recommended because of the high risk of ischemic complications. The anatomical differences in the gyrencephalic swine brain might help to elucidate why prior efforts at experimental production of venous infarction through SSS thrombosis have generally been unsuccessful. Two successful alternatives for inducing infarctions after cerebral thrombosis in swine are bilateral occlusion or massive injection of prothrombotic material into the jugular vein [34] and the occlusion of two adjacent bridging veins to produce a focal venous infarction [40]. SDs can result from hypoxia and ischemia following arterial occlusion. After cerebral ischemia, SDs frequently occur experimentally and clinically, originating from compromised tissue in the ischemic penumbra [41, 42], triggered by fluctuations in metabolic supply [43–45]. In rat retina, SDs have been successfully induced post-photothrombosis after occlusion of arterioles and venules [46], though arterial pathology may mask venous injury effects. SDs have also been observed following cerebral venous occlusions in lissencephalic mouse brains [47], and after cortical vein occlusion in rat brains [40, 48]. However, the present study shows that SDs can be induced in the gyrencephalic brain following venous occlusion by clipping the middle third of the SSS. Unlike our arterial stroke model [9–11], this study found fewer SDs and no infarction. SDs occurred primarily in the first experimental hour, while in the arterial model, SD incidence increased over time, often in clusters. The mean incidence was 6.4 SDs/h (± 2.9) in the arterial model [11], compared to 0.3 SDs/h (± 0.7) after venous occlusion. These results highlight differences in SD dynamics between arterial and venous occlusion, suggesting venous occlusion leads to a different mechanism, possibly related to impaired venous drainage rather than severe ischemia.

Physiologically, it is recognized that sinus thrombosis raises venous pressure, hindering CSF absorption, leading to cerebral edema and increased ICP [49–51]. We hypothesize that SSS occlusion causes reverse flow and vascular hypertension sufficient to induce SDs. While venous ischemia is a concern, our IOS findings suggest this is unlikely, as the lack of infarction and specific hemodynamic signatures do not match ischemic patterns. However, this cannot be fully excluded, and further studies are needed. In comparison to our normoxic and arterial ischemic swine models, we observed different hemodynamic SD responses in the IOS imaging, with less heterogeneity. This study identified four distinct SD morphologies, characterized primarily by peak hyperemia (component III), either alone or with late hyperemia (component IV). These differences are probably linked to tissue microenvironment variations, such as oxygenation, metabolic activity, and vascular tone, affecting how vascular compartments respond to SDs [52]. Venous occlusion resulted in predominantly hyperemic hemodynamic responses, likely driven by intravascular perfusion pressure, which influenced both the intensity and duration of these hyperemic phases [53]. In this case, we did not observe perfusion pressure changes after clipping, except in one animal. Although there is limited research on how perfusion pressure affects SDs, our findings are consistent with ischemia studies, which report oligemic responses in areas where cerebral perfusion is reduced and hyperemic responses where cerebral perfusion remains within normal ranges [54], highlighting the critical role of vascular pressure in determining hemodynamic outcomes. Future research is essential to clarify how intravascular pressure variations affect SDs, aiding the development of targeted therapies. These studies could help reduce the impact of SDs in venous occlusion, improving clinical outcomes and advancing our understanding of cerebrovascular pathophysiology.

Movement artifacts limited robust data extraction from LSCI, but the congruence between IOS and LSCI data strengthens our findings. The patterns of OxyHb and deOxyHb responses during SD suggest near-normal perfusion, supported by the observation that the oxygen supply-demand mismatch in brain tissue caused by SD did not manifest strong hypoperfusion or ischemic responses, which is a significant insight into the underlying mechanisms. In arterial ischemia, initial hypoperfusion (component II) and post-SD oligemia (component V) are linked to increased O_2_ extraction and reduced O_2_ saturation [55]. However, in the SSS occlusion model, O_2_ availability seemed adequately compensated, as indicated by predominant hyperemic reactions (components III and IV). This suggests SDs can occur without significant ischemia, contrasting typical arterial occlusion models where SDs are tied to ischemic conditions. Hyperemic responses in our model are likely associated with vascular collateralization, which maintained adequate oxygenation during SD [56]. To our knowledge, no prior studies in gyrencephalic brains have documented SDs in the absence of ischemia, chemical insult, or blood presence, underscoring the uniqueness of our approach. While minor red cell extravasation might be missed, our results suggest SDs can occur independently of these factors, providing new insights into SDs under altered cerebral venous drainage.

### Study limitations

While this study offers valuable insights, several limitations must be acknowledged. First, prospective longitudinal research is crucial to fully elucidate the sequence of pathophysiological events leading to SD following venous occlusion, as well as to establish a definitive link to venous infarction. In particular, due to ethical considerations, brain tissue from the swine models was collected at an early stage. This may have occurred before thrombosis or venous infarction had sufficient time to develop, limiting our ability to conclusively determine whether the model would have resulted in venous infarction at later time points. As a result, caution is advised when interpreting the potential of this model to induce venous infarction. Nevertheless, this study was exploratory in nature, focusing primarily on the immediate consequences of SSS occlusion and the occurrence of SD. Second, the clipping technique used, although effective for our study’s aims, has certain inherent drawbacks. Specifically, it involves significant cortical exposure and manipulation of the dura mater, which may inadvertently compromise smaller bridging veins and disrupt glymphatic drainage. These factors may have influenced the results, introducing complexities in interpreting venous drainage pathways following occlusion. Third, another limitation is the absence of an untreated control group in this study. However, this limitation does not critically impact the primary objective, which was to explore the SD hemodynamic response after SSS occlusion. This decision was based on prior data from our group, which demonstrated that SDs do not occur spontaneously in well-perfused swine brains post-craniotomy in control groups [11]. In previous studies, SDs were only elicited after preconditioning with KCl [12–14, 57]. Additionally, the lack of SDs detected in ECoG and IOS imaging recordings prior to SSS occlusion further justifies the exclusion of an untreated control group. Therefore, the inclusion of a control group in this study would not have provided additional meaningful data. Thus, future studies with larger sample sizes, longitudinal follow-up, and the inclusion of control animals will be essential to validate these observations and further elucidate the pathophysiological mechanisms at play.

## Conclusion

Our investigation has elucidated, with unprecedented clarity, that SDs can arise spontaneously after occlusion of the SSS within the gyrencephalic porcine brain. Crucially, this phenomenon manifests independently of venous infarction, indicating a novel pathophysiological process. The implications of our findings are for extensive validation through additional research utilizing both in vivo animal models and rigorous clinical studies to enhance our understanding and inform therapeutic strategies for SSS occlusive disorders.

## Electronic supplementary material

Below is the link to the electronic supplementary material.

Online Resource 1: Video 1: Visualization of SD progression via IOS imaging. The provided video captures the propagation dynamics of a spreading depolarization (SD) event across the cortical regions of both hemispheres post-occlusion in the swine model. Initiation is observed at the site of surgical intervention on the superior sagittal sinus (SSS) and progresses anteriorly, tracing the complex gyral patterns of the parietal and frontal lobes. Intrinsic optical signal (IOS) imaging is utilized to map the hemodynamic responses accompanying the SD, with regions of increased cerebral blood volume (CBV) indicated by a red hue and decreased CBV by a purple hue, which can be quantitatively tracked via the adjacent colorimetric scale

Online Resource 2: Video 2a-c Expansion of SD through the cerebral cortex in LSCI recordings. Videos 2a-c present sequential LSCI segments, utilizing the MoorO2Flo system to elucidate cerebral blood flow (CBF) and hemoglobin oxygenation changes corresponding to spreading depolarization (SD) events. Four regions of interest (ROIs) are demarcated on the cerebral cortex to meticulously monitor perfusion and oxygenation shifts post-SD. In **Video 2a**, the progression of SD across the right hemisphere’s parietal to frontal cortex, approximately nine minutes post-SSS occlusion, is captured, with transient hyperemia evidenced by an upsurge in the laser speckle contrast and oxygen imaging (LSCI) signal intensity. Subsequent **Videos 2b and 2c** delineate the SD’s influence on oxygenation, with deoxyhemoglobin (deOxyHb) and oxyhemoglobin (OxyHb) variations, respectively, conveyed via alterations in LSCI signal intensity. Notably, an increase in signal intensity denotes a decrease in deOxyHb while augmented OxyHb during the SD event

## Data Availability

No datasets were generated or analysed during the current study.
